# Presenting features of neuroblastoma with spinal canal invasion. A prospective study of the International Society of Pediatric Oncology Europe - Neuroblastoma (SIOPEN)

**DOI:** 10.3389/fped.2022.1023498

**Published:** 2022-10-10

**Authors:** Stefania Sorrentino, Shifra Ash, Riccardo Haupt, Dominique Plantaz, Isabelle Schiff, Barbara Hero, Thorsten Simon, Denis Kachanov, Tatyana Shamanskaya, Katheljine Kraal, Annemieke Littooij, Alexsandra Wieczoreck, Walentyna Balwierz, Geneviève Laureys, Catherine Trager, Fiammetta Sertorio, Giovanni Erminio, Martina Fragola, Maja Beck Popovic, Bruno De Bernardi, Toby Trahair

**Affiliations:** ^1^Paediatric Oncology Unit, IRCCS Istituto Giannina Gaslini, Genova, Italy; ^2^Joan and Sanford Weill Paediatric Haematology Oncology and Bone Marrow Transplantation Division, Ruth and Bruce Rappaport Faculty of Medicine, Technion Israel Institute of Technology, Haifa, Israel; ^3^DOPO Clinic, IRCCS Istituto Giannina Gaslini, Genova, Italy; ^4^Department de Paediatrics, Hôpital Couple Enfants, CHU Grenoble, Grenoble, France; ^5^Department of Paediatric Haematology and Oncology, Children's Hospital, University of Cologne, Cologne, Germany; ^6^Department of Clinical Oncology, Dmitry Rogachev National Medical Research Center of Paediatric Haematology, Oncology and Immunology, Moscow, Russia; ^7^Princess Màxima Centre for Paediatric Oncology, Utrecht, Netherlands; ^8^Paediatric Oncology and Haematology Department, Institute of Paediatrics, Jagiellonian University, Krakow, Poland; ^9^Department of Paediatric Haematology-Oncology, Prinses Elisabeth Kinderziekenhuis, University Hospital, Gent, Belgium; ^10^Women's and Childrens Health, Karolinska Institutet, Stockholm, Sweden; ^11^Radiology Unit, IRCCS Istituto Giannina Gaslini, Genova, Italy; ^12^Scientific Directorate, IRCCS Istituto Giannina Gaslini, Genova, Italy; ^13^Centre Hospitalier Universitaire Vaudois, Unité d'Hémato-Oncologie Pédiatrique, Lausanne, Switzerland; ^14^Kids Cancer Center, Sydney Children's Hospital, Randwick, NSW, Australia; ^15^Children's Cancer Institute, Lowy Cancer Research Centre, University of New South Wales, Kensington, NSW, Australia; ^16^School of Women's and Children's Health, University of New South Wales, Kensington, NSW, Australia

**Keywords:** neuroblastoma, spinal canal invasion, epidural spinal cord compression, long-term disabilities, pediatric tumors

## Abstract

**Introduction:**

Between 5 and 15% of children with neuroblastoma (NB) present with or develop spinal canal invasion (SCI). The majority of these children have symptoms of epidural compression of spinal cord and/or spinal nerves. Treatment of NB-SCI is considered an emergency but its modalities are not yet well-established. Independently of treatment, NB-SCI may result in significant long-term disabilities. We report on the first prospective study of NB-SCI focused on presenting characteristics of both symptomatic and asymptomatic patients and correlation between SCI-related symptoms and imaging features.

**Materials and methods:**

This SIOPEN prospective NB-SCI study opened in June 2014. Patient data including SCI symptoms evaluated by standardized measures and spinal cord imaging studies were collected for each patient. For the purpose of this study data entry was locked on July 2021.

**Results:**

Of the 208 NB-SCI patients registered, 196 were evaluable for this analysis of whom 67% were symptomatic and 33% asymptomatic. Median age was 11 months. The thorax was the commonest primary tumor site. The median intervals between initial symptoms and diagnosis and between first medical visit and diagnosis were 14 and 3 days, respectively. The was no statistical difference in frequency of presenting characteristics between symptomatic and asymptomatic patients. Presenting features of NB-SCI patients differed from other NBs for older median age, prevalence of thoracic vs. abdominal primary site, prevalence of localized vs. metastatic disease and lower incidence of MYCN gene amplification. The most common SCI features were motor deficit in the younger and pain in the older patients that correlated on imaging with both transverse and longitudinal extent but not with the level of intraspinal tumor. Spinal cord T2-hyperintensity was more frequently detected in symptomatic patients (not significant).

**Conclusion:**

This prospective study confirms that children with NB-SCI differ from NBs without SCI. Compared to previous studies, it provides more detailed information regarding presenting symptoms, time intervals between SCI symptoms, medical visit and diagnosis, and correlations between symptoms and imaging features.

## Introduction

Neuroblastoma (NB) originates from the primitive sympathetic cells of the adrenal medulla and paraspinal sympathetic ganglia and represents the commonest extracranial neoplasm of childhood (1). As NB grows close to the spine, it may infiltrate the intervertebral foramina, invade the spinal canal (SCI) and compress the spinal cord and/or nerve roots (2). Spinal canal invasion (SCI), defined as the tumor growth through one or more neural foramina extending into the spinal canal (3), is commonly symptomatic (4–6). Although this condition has been recognized since the early 1950's (7), its natural history is not clearly defined, due to heterogeneity and retrospective nature of studies (4, 8–14). NB-SCI accounts for 5–15% of all NBs (5, 6, 8–14) and are thought to have favorable presenting features and better survival (8–14). However, NB-SCI patients are at high risk of developing significant long-term disabilities (11, 15–17). Thus, NB-SCI patients require urgent multidisciplinary evaluation, prompt initiation of treatment and focused follow-up (5, 6, 18). SCI treatment includes neurosurgery (19), chemotherapy (20), and/or radiotherapy (21), all effective in relieving symptoms, although there is no consensus on treatment approach (5, 6, 14, 22). To increase and refine the information about presenting features, treatment, clinical course, long-term disabilities and outcome of NB-SCI, the SIOPEN launched the prospective NB-SCI Study in 2014. In this report we have described the NB-SCI presenting characteristics of these patients and correlated them with imaging features.

## Materials and methods

NB-SCI is a prospective, observational, multinational study which opened in June 2014 (ClinicalTrials.Gov Identifier: NCT02559804) with the aim of collecting data on NB patients younger than 18 years at diagnosis with clinical and/or imaging features of SCI. The only exclusion criterion was the administration of anti-cancer treatments in the 6 preceding months. The study primary objectives were (i) to describe the natural history of NB presenting with SCI, and (ii) evaluate the combined effects of different risk factors on the eventual functional outcome. Secondary study objectives were (i) to correlate pathologic and biological characteristics with clinical features, response to therapy and sequelae; (ii) describe diagnostic and therapeutic approaches adopted in the participating centers; (iii) increase the awareness of pediatricians about NB-SCI; and (iv) develop common guidelines for the management of these patients. The study end points were: (i) response to therapy; (ii) long-term prevalence and severity of late sequelae; (iii) occurrence of relapse; and (iv) survival. Data were collected using case report forms which included patients' demographic features, diagnostic work-up including histopathology (23), biology and disease staging (24, 25), type and severity of SCI-related symptoms, intervals between symptom onset and diagnosis and first medical visit and diagnosis of SCI, SCI imaging features, treatment for SCI and underlying NB, follow-up and long-term disabilities. The NB-SCI study did not provide recommendations regarding management of NB and SCI, but only recorded the treatment used by the responsible physicians.

SCI-related symptoms were recorded using standardized measures including the ASIA Impairment Scale (26) for motor deficit and the CTCAE version 4.0 (27) for back-radicular pain, spine deformities, sensory deficits, bladder and bowel dysfunctions and dyspnoea. The following exceptions were made: (i) in patients younger than 36 months pain was assessed using the FLACC score (28); (ii) bladder and bowel functions were considered not assessable in patients younger than 24 months and were defined as normal or abnormal in patients 24–35 months old. Severity scores of the different scales were harmonized in four categories (Table 1).

**Table 1:** Harmonization of severity scores for symptom grading.

	**Score method**	**Severity score**			
**Symptom**		**None**	**Mild**	**Moderate**	**Severe**
**Motor deficit**					
Any age	ASIA	0	1	2	3
**Pain**					
Age 0–35 mos	FLACC	0	1–3	4–6	7–10
Age ≥ 36 mos	CTCAE	0	1	2	3
**Dyspnea**					
Any age	CTCAE	0	1	2	3–5
**Sensory deficit**					
Any age	CTCAE	0	1	2	3
**Sphincter dysfunction**					
Age 0–23 mos	Clinical	Not assessable
Age 24–35 mos	Clinical	Normal	Mild	Moderate	Severe
Age ≥ 36 mos	CTCAE	0	1	2	3–4
**Spine deformities**					
Any age	CTCAE	0	1	2	3

Magnetic resonance imaging (MRI) and computed tomography (CT) were used to document the following SCI features: (i) degree of transverse spinal canal invasion, quantified as <33%, between 33 and 66% and >66% (3); (ii) longitudinal intraspinal extension, expressed as the number of vertebrae between the upper and lower SCI limits, and categorized as 1–3, 4–6, >6 vertebrae based on their IQR distribution; (iii) intraspinal level, defined as cervical, thoracic, abdominal or pelvic; (iv) T2 weighted hyperintensity of spinal cord related to the presence of spinal cord oedema (29).

The study was approved by the Ethics Committee of participating institutions and/or national NB groups. All participants or their parents/legal guardians gave consent to participate in this study. The data lock for this report was July 2021.

### Statistics

Descriptive statistics were reported as absolute frequencies and percentages for qualitative variables and as median values with their IQR and minimum and maximum value for the quantitative ones. Wilcoxon-Mann-Whitney's test was used to compare median. Pearson chi-square and Fisher exact test, when appropriate, were applied to compare proportions between groups and a *P*-value < 0.05 was considered significant. All analyses were performed by the statistical package Stata (version 13.0, Stata Corporation, College Station, TX).

## Results

### Patient cohort

Between July 2014 and July 2021, 208 eligible patients were registered from institutions of 16 national groups (Appendix). Twelve were excluded for incomplete data leaving 196 evaluable of whom 132 (67%) had SCI-related symptoms and 64 (33%) were asymptomatic. Eleven patients (5%) had coexisting medical conditions: congenital cardiomyopathies and musculoskeletal abnormalities (three cases each), unspecified brain abnormalities and motor delay (two cases each) and neurofibromatosis type 1 (one case). Two patients had opsomyoclonus syndrome. The presenting features of the study population are listed in Table 2.

**Table 2:** Presenting features of 196 NB-SCI patients.

**Feature**	**All patients**	**Symptomatic patients**	**Asymptomatic patients**	***P***
	***N* (%)**	***N* (%)**	***N* (%)**	
	**196 (100)**	**132 (67)**	**64 (33)**	
**Male/female ratio**	0.82	0.83	0.78	0.822
**Age (months) at SCI imaging**				
Median (IQR)	11 (4–28)	10 (4–25)	12.5 (5–32.5)	0.326^$^
0–35	156 (80)	108 (82)	48 (75)	0.267
0–23	137 (88)	97 (90)	40 (83)	
24–35	19 (12)	11 (10)	8 (17)	
≥36	40 (20)	24 (18)	16 (25)	
**Time of enrolment**				0.038*
Diagnosis	191 (97)	131 (99)	60 (94)	
Relapse	5 (3)	1 (1)	4 (6)	
**Symptom-diagnosis interval (days)**				-
Median (IQR)	-	14 (5–34)	-	
0–6	-	37 (28)	-	
7–30	-	55 (42)	-	
31–60		23 (18)		
>60	-	16 (12)	-	
**First visit-diagnosis interval (days)**				
Median (IQR)	3 (1–10)	3 (1–9)	3 (1–10)	
0–6	130 (67)	89 (68)	41 (65)	0.271
7–30	42 (22)	30 (23)	12 (19)	
31–60	15 (8)	9 (7)	6 (10)	
>60	6 (3)	2 (2)	4 (6)	
Primary site**				0.184
Neck	17 (9)	12 (9)	5 (8)	
Thorax	97 (49)	72 (55)	25 (39)	
Abdomen (including 10 adrenal = 8%)	63 (32)	38 (29)	25 (39)	
Pelvis	12 (6)	7 (5)	5 (8)	
Double site	7 (4)	3 (2)	4 (6)	
**Site of surgery**				0.001*
Extraspinal	142 (74)	85 (66)	56 (90)	
Intraspinal	41 (21)	37 (29)	4 (6)	
Extra+intraspinal	4 (2)	3 (2)	1 (2)	
Bone marrow	5 (3)	4 (3)	1 (2)	
**Histopathology (*****n*** **=** **192)**		130	62	0.395*
Malignant	171 (89)	118 (91)	53 (85)	
NB poorly differentiated	128 (75)	88 (75)	40 (75)	
NB other	43 (25)	30 (25)	13 (25)	
Benign	21 (11)	12 (9)	9 (15)	
GNB intermixed	6 (29)	4 (33)	2 (22)	
GN	15 (71)	8 (67)	7 (78)	
**Biology**				
MYCN (*n* = 183)				0.121*
Normal	154 (93)	118 (95)	52 (88)	
Amplified	13 (7)	6 (5)	7 (12)	
Chromosome aberration (*n* = 129*)*				0.302
None	47 (36)	32 (35)	15 (40)	
Numerical	53 (41)	36 (39)	17 (46)	
Segmental ± numerical	29 (23)	24 (26)	5 (14)	
**INRG stage**				0.058*
L1^∧^	8 (4)	4 (3)	4 (6)	
L2^∧∧^	121 (62)	78 (59)	43 (67)	
M	51 (26)	35 (27)	16 (25)	
MS	16 (8)	15 (11)	1 (2)	

* Fisher exact test.

$ Mann-Whitney test.

** Neck includes cervico-thoracic. Thorax includes thoraco-abdominal. Abdomen includes abdomino-pelvic.

∧ Includes three benign tumors.

∧∧ Includes 18 benign tumors.

The male to female ratio was 0.82. Median age was 11 months (IQR 4–28; range, 0–201) with 80% of patients younger than 36 months. One-hundred ninety-one (97%) were enrolled at diagnosis and 5 at relapse. The more frequent primary tumor sites were thorax (49%) and abdomen (32% including 8% adrenals), followed by neck (9%) and pelvis (6%). Seven patients (4%) had two primary sites with SCI documented in both sites in two.

In the 132 symptomatic patients, the median interval between SCI symptoms and diagnosis was 14 days (IQR, 5–34; range, 0–705) and was between 0 and 6 days in 37 (28%) and above 60 days in 16 (12%). These latter included 9/171 (5%) of patients with NB and 7/21(33%) with benign histology (data not shown). The median interval between the first medical visit and diagnosis in symptomatic patients was 3 days (IQR, 1–9; range, 0–71) and was between 0 and 6 days in 89 (68%) and above 60 days in 2 (2%), with similar figures observed both in the entire cohort and asymptomatic patients.

The tumor diagnosis was based on surgical specimens in 98% of patients. In asymptomatic patients surgery was more frequently performed on the extraspinal component (*P* < 001). Only four patients did not undergo surgery: the diagnosis was based on clinical and radiological data in 2 and on the initial histopathology in the other two.

Tumor histopathology showed malignant features in 171 patients (89%), mostly poorly differentiated NB, while the remaining 21 (11%) had benign features: ganglioneuroblastoma intermixed in 6 and ganglioneuroma in 15. Biological features were mostly evaluated in malignant NB: MYCN gene status was assayed in 183 cases and found amplified in 13 (7%) and segmental chromosomal abnormalities (SCA) were assayed in 129 tumors with 29 (23%) having segmental aberrations.

The was no statistical difference for all these features between symptomatic and symptomatic patients.

### Imaging studies

Spinal MRI was performed in 193 patients (98%), 59 of them were also evaluated with CT, three patients underwent CT examination only (Table 3). The transverse degree of SCI was evaluated in 187 children and was <33% in 23%, between 33 and 66% in 28% and >66% in 49%. It directly correlated with presence of symptoms, with 63% of symptomatic patients having a degree >66%, and 48% of those asymptomatic having a SCI degree <33% (*P* < 0.001). The longitudinal intraspinal extension was measured in 194 patients and involved a median of 4 vertebrae (IQR 3–6), with longitudinal extent of 1–3 vertebrae in 30%, between 4 and 6 in 51%, and 7–15 in 19%. The longitudinal extent was greater in symptomatic patients either if calculated as a continuous or categorical variable (*P* < 0.001). The intraspinal tumor was documented at cervical level in 6% of patients, thoracic in 44%, abdominal in 41% and pelvic in 9% without a difference between symptomatic and asymptomatic patients. The transverse degree of SCI increased with longitudinal tumor extent (*P* < 0.001) but not with intraspinal SCI level (Table 4). T2 hyperintensity of the spinal cord was detected in 44% of the 172 tested cases, with 48% of symptomatic showing this feature vs. 35% of those asymptomatic (not significant).

**Table 3:** Neuroradiologic features of NB-SCI patients.

	**Tested patients**	**Symptomatic patients**	**Asymptomatic patients**	***P***
	***N* (%)**	***N* (%)**	***N* (%)**	
	**196 (100)**	**132 (67)**	**64 (33)**	
**Type of imaging**				0.322*
MRI only	134 (68)	93 (70)	41 (64)	
MRI + CT	59 (30)	38 (29)	21 (33)	
CT only	3 (2)	1 (1)	2 (3)	
**Transverse degree of SCI (*****n*** **=** **187)**				<0.001
<33%	43 (23)	14 (11)	29 (48)	
33–66%	52 (28)	33 (26)	19 (31)	
>66%	92 (49)	79 (63)	13 (21)	
**Longitudinal intraspinal extension (*****n*** **=** **194)**				
Involved vertebrae, median (IQR)	4 (3–6)	5 (4–6)	3 (3–5)	<0.001^$^
1–3	59 (30)	26 (20)	33 (52)	
4–6	98 (51)	74 (57)	24 (37)	<0.001
>6	37 (19)	30 (23)	7 (11)	
**Intraspinal level**^**#**^ **(*****n*** **=** **194)**				0.160*
Cervical	11(6)	9 (7)	2 (3)	
Thoracic	85 (44)	59 (45)	26 (41)	
Abdominal	80 (41)	54 (42)	26 (41)	
Pelvic	18 (9)	8 (6)	10 (15)	
**MRI T2 hyperintensity (*****n*** **=** **172)**				0.113
Yes	75 (44)	55 (48)	20 (35)	
No	97 (56)	60 (52)	37 (65)	

# In cases of SCI encompassing two contiguous levels and double primary, the location with the greater percentage of vertebrae involved was considered.

* Fisher exact test.

$ Mann-Whitney test.

**Table 4:** Transverse degree of SCI in relation to longitudinal intraspinal extension and intraspinal level.

	**Patients**	**Transverse degree**			
		**<33%**	**33–66%**	**>66%**	***P***
	***N* (%)**	***N* (%)**	***N* (%)**	***N* (%)**	
**Longitudinal intraspinal extension**	186	43	51	92	
1–3 vertebrae	56 (30)	22 (51)	20 (39)	14 (15)	<0.001
4–6 vertebrae	93 (50)	18 (42)	22 (43)	53 (58)	
>6 vertebrae	37 (20)	3 (7)	9 (18)	25 (27)	
**Intraspinal level**	186	43	51	92	
Cervical	11 (6)	2 (5)	1 (2)	8 (9)	0.211*
Thoracic	81 (44)	16 (37)	27 (53)	38 (41)	
Abdominal	77 (41)	18 (42)	18 (35)	41 (45)	
Pelvic	17 (9)	7 (16)	5 (10)	5 (5)	

* Fisher exact test.

### Symptoms

**Figure 1:**
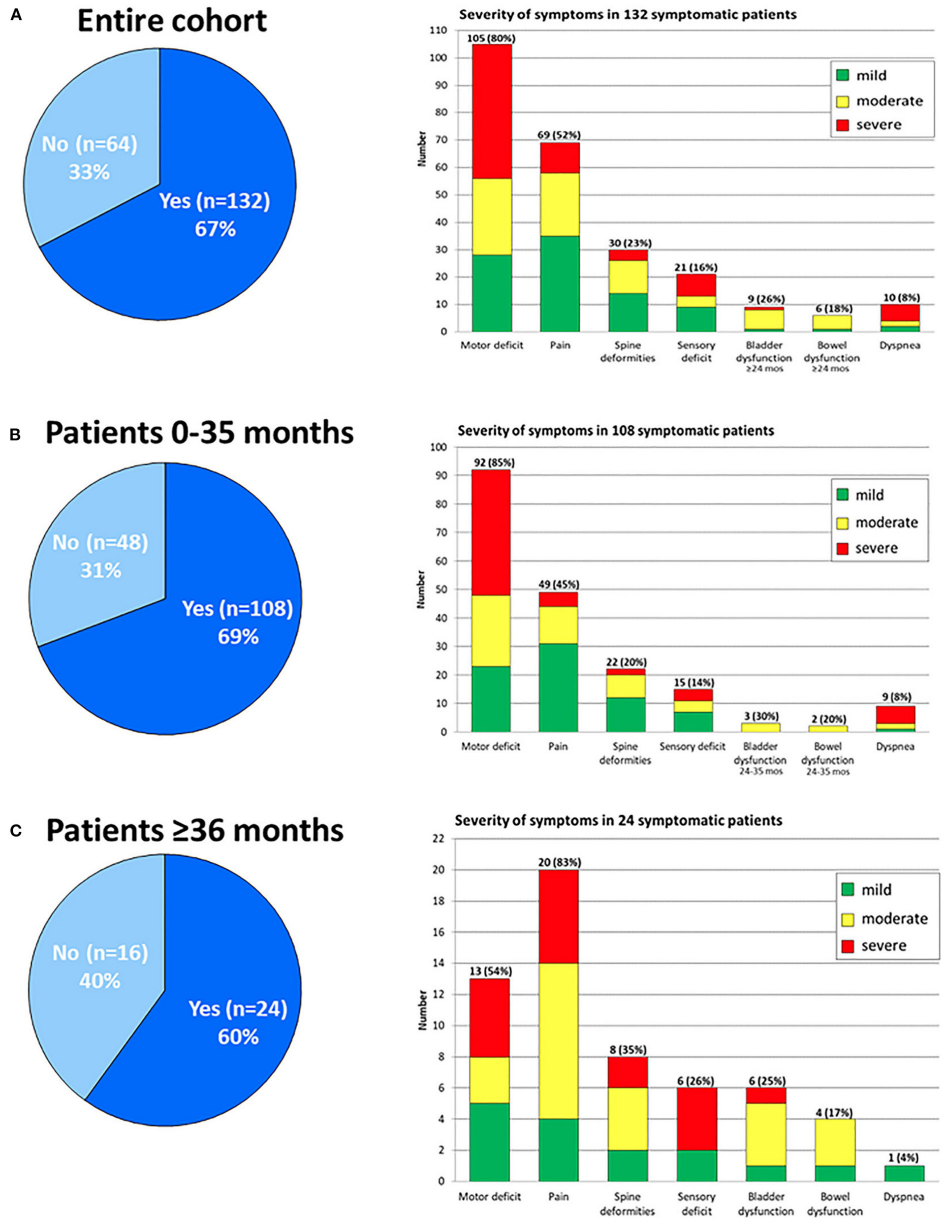
Presence of SCI-related symptoms in the entire patient cohort [**(A)**, left]. Frequency, types and severity scores of symptoms in symptomatic patients [**(A)**, right]. Same for the 0–35 months old **(B)** and ≥36 months old patients **(C)**.

Figure 1 and Table 5 depict the frequency, type and severity of symptoms in the 132 symptomatic patients (Figure 1A), and in patients stratified by age at diagnosis (Figures 1B,C), whereas the correlation between symptoms and imaging features are detailed in Table 6. A total of 250 symptoms were reported with a median of two symptoms per patient (range, 1–6) without differences by age.

**Table 5:** SCI symptoms at presentation of 132 symptomatic patients in relation to age.

	**Presence**			**Severity**			
	**No**	**Yes**	***P***	**Mild**	**Moderate**	**Severe**	***P***
**Motor deficit (*****n*** **=** **132)**	27 (20)	105 (80)		28 (27)	28 (27)	49 (46)	
0–35 mos (*n* = 108)	16 (15)	92 (85)	0.001	23 (24)	25 (28)	44 (48)	0.647*
≥36 mos (*n* = 24)	11 (46)	13 (54)		5 (38)	3 (24)	5 (38)	
**Pain (*****n*** **=** **132)**	63 (48)	69 (52)		35 (51)	23 (33)	11 (16)	
0–35 mos (n = 108)	59 (55)	49 (45)	0.001	31 (63)	13 (27)	5 (10)	0.003*
≥36 mos (*n* = 24)	4 (17)	20 (83)		4 (20)	10 (50)	6 (30)	
**Spine deformities (*****n*** **=** **131)**	101 (77)	30 (23)		14 (47)	12 (40)	4 (13)	
0–35 mos (*n* = 108)	86 (80)	22 (20)	0.135	12 (55)	8 (36)	2 (9)	0.228*
≥36 mos (*n* = 23)	15 (65)	8 (35)		2 (25)	4 (50)	2 (25)	
**Sensory deficit (*****n*** **=** **130)**	109 (84)	21 (16)		9 (43)	4 (19)	8 (38)	
0–35 mos (*n* = 107)	92 (86)	15 (14)	0.154	7 (46)	4 (27)	4 (27)	0.221*
≥36 mos (*n* = 23)	17 (74)	6 (26)		2 (33)	0	4 (67)	
**Bladder dysfunction (*****n*** **=** **34)**	25 (74)	9 (26)		1 (11)	7 (78)	1 (11)	
24–35 mos (*n* = 10)	7 (70)	3 (30)	1.000*	0	3 (100)	0	1.000*
≥36 mos (*n* = 24)	18 (75)	6 (25)		1 (17)	4 (66)	1 (17)	
**Bowel dysfunction (*****n*** **=** **34)**	28 (82)	6 (18)		1 (17)	5 (83)	0	
24–35 mos (*n* = 10)	8 (80)	2 (20)	1.000*	0	2 (100)	0	1.000*
≥36 mos (*n* = 24)	20 (83)	4 (17)		1 (25)	3 (75)	0	
**Dyspnea (*****n*** **=** **131)**	121 (92)	10 (8)		2 (20)	2 (20)	6 (60)	
0–35 mos (*n* = 108)	99 (92)	9 (8)	1.000*	1 (11)	2 (22)	6 (67)	0.400*
≥36 mos (*n* = 23)	22 (96)	1 (4)		1 (100)	0	0	

**Table 6:** SCI symptoms in relation to imaging features.

**Symptom**	**Presence**			**Severity**			
	**No**	**Yes**	***P***	**Mild**	**Moderate**	**Severe**	***P***
**Motor deficit**							
**Transverse degree of SCI**							
Tested patients (*n* = 187)	86 (46)	101 (54)	<0.001	27 (27)	27 (27)	47 (46)	0.019*
<33%	31 (72)	12 (28)		5 (42)	6 (50)	1 (8)	
33–66%	29 (56)	23 (44)		8 (35)	6 (26)	9 (39)	
>66%	26 (28)	66 (72)		14 (21)	15 (23)	37 (56)	
**Longitudinal intraspinal extension**							
Tested patients (*n* = 194)	89 (46)	105 (54)	0.001	28 (27)	28 (27)	49 (46)	0.037
1–3 vertebrae	39 (66)	20 (34)		11 (55)	3 (15)	6 (30)	
4–6 vertebrae	39 (40)	59 (60)		12 (20)	17 (29)	30 (51)	
>6 vertebrae	11 (30)	26 (70)		5 (19)	8 (31)	13 (50)	
**Intraspinal level**							
Tested patients (*n* = 194)	89 (46)	105 (54)	0.002*	28 (27)	28 (27)	49 (46)	0.654*
Cervical	2 (18)	9 (82)		3 (33)	2 (22)	4 (45)	
Thoracic	38 (45)	47 (55)		11 (23)	14 (30)	22 (47)	
Abdominal	34 (43)	46 (57)		12 (26)	11 (24)	23 (50)	
Pelvic	15 (83)	3 (17)		2 (67)	1 (33)	0	
**Pain**							
**Transverse degree of SCI**							
Tested patients (*n* = 187)	121 (65)	65 (35)	0.031	34 (51)	21 (32)	11 (17)	0.863*
<33%	35 (81)	8 (19)		3 (37)	3 (37)	2 (26)	
33–66%	30 (58)	22 (42)		12 (55)	6 (27)	4 (18)	
>66%	56 (61)	36 (39)		19 (53)	12 (33)	5 (14)	
**Longitudinal intraspinal extension**							
Tested patients (*n* = 194)	126 (65)	68 (35)	0.676	34 (50)	23 (34)	11 (16)	0.365*
1–3 vertebrae	41 (69)	18 (31)		7 (39)	7 (39)	4 (22)	
4–6 vertebrae	62 (63)	36 (37)		17 (47)	14 (39)	5 (14)	
>6 vertebrae	23 (62)	14 (38)		10 (71)	2 (14)	2 (14)	
**Intraspinal level**							
Tested patients (*n* = 194)	126 (65)	68 (35)	0.938	34 (50)	23 (34)	11 (16)	0.198
Cervical	7 (70)	3 (30)		1 (25)	3 (75)	0	
Thoracic	57 (67)	28 (33)		13 (46)	12 (43)	3 (11)	
Abdominal	50 (63)	30 (37)		18 (60)	6 (20)	6 (20)	
Pelvic	12 (67)	6 (33)		2 (33)	2 (33)	2 (33)	
**Spine deformities**							
**Transverse degree of SCI**							
Tested patients (*n* = 187)	157 (84)	30 (16)	0.153	14 (47)	12 (40)	4 (13)	0.742*
<33%	40 (93)	3 (7)		1 (33)	2 (67)	0	
33–66%	41 (79)	11 (21)		4 (36)	5 (46)	2 (18)	
>66%	76 (83)	16 (17)		9 (56)	5 (31)	2 (13)	
**Longitudinal intraspinal extension**							
Tested patients (*n* = 193)	163 (84)	30 (16)	0.023	14 (47)	12 (40)	4 (13)	0.116*
1–3 vertebrae	55 (95)	3 (5)		2 (67)	1 (33)	0	
4–6 vertebrae	80 (82)	18 (18)		5 (28)	9 (50)	4 (22)	
>6 vertebrae	28 (76)	9 (24)		7 (78)	2 (22)	0	
**Intraspinal level**							
Tested patients (*n* = 193)	163 (84)	30 (16)	0.324	14 (47)	12 (40)	4 (13)	0.345*
Cervical	9 (82)	2 (18)		2 (100)	0	0	
Thoracic	71 (84)	14 (16)		8 (57)	5 (36)	1 (7)	
Abdominal	66 (83)	14 (17)		4 (29)	7 (50)	3 (21)	
Pelvic	17 (100)	0		0	0	0	
**Sensory deficit**							
**Transverse degree of SCI**							
Tested patients (*n* = 186)	165 (89)	21 (11)	0.258	9 (43)	4 (19)	8 (38)	1.000*
<33%	41 (95)	2 (5)		1 (50)	0	1 (50)	
33–66%	46 (88)	6 (12)		3 (50)	1 (17)	2 (33)	
>66%	78 (86)	13 (14)		5 (39)	3 (22)	5 (39)	
**Longitudinal intraspinal extension**							
Tested patients (*n* = 192)	171 (89)	21 (11)	0.180*	9 (43)	4 (19)	8 (38)	0.220*
1–3 vertebrae	55 (95)	3 (5)		0	0	3 (100)	
4–6 vertebrae	85 (88)	12 (12)		5 (42)	3 (25)	4 (33)	
>6 vertebrae	31 (84)	6 (16)		4 (66)	1 (17)	1 (17)	
**Intraspinal level**							
Tested patients (*n* = 192)	171 (89)	21 (11)	0.876	9 (43)	4 (19)	8 (38)	1.000*
Cervical	10 (91)	1 (9)		1 (100)	0	0	
Thoracic	75 (89)	9 (11)		3 (33)	3 (33)	3 (33)	
Abdominal	70 (87)	10 (13)		4 (40)	3 (30)	3 (30)	
Pelvic	16 (94)	1 (6)		1 (100)	0	0	
**Bladder dysfunction**							
**Transverse degree of SCI**							
Tested patients (*n* = 54)	45 (83)	9 (17)	0.345*	1 (11)	7 (78)	1 (11)	1.000*
<33%	16 (94)	1 (6)		0	1 (100)	0	
33–66%	13 (81)	3 (19)		0	3 (100)	0	
>66%	16 (76)	5 (24)		1 (20)	3 (60)	1 (20)	
**Longitudinal intraspinal extension**							
Tested patients (*n* = 57)	48 (84)	9 (16)	0.084*	1 (11)	7 (78)	1 (11	1.000*
1–3 vertebrae	23 (96)	1 (4)		0	1 (100)	0	
4–6 vertebrae	20 (77)	6 (23)		1 (17)	4 (66)	1 (17)	
>6 vertebrae	5 (71)	2 (29)		0	2 (100)	0	
**Intraspinal level**							
Tested patients (*n* = 57)	48 (84)	9 (16)	0.537*	1 (11)	7 (78)	1 (11)	0.222*
Cervical	1 (50)	1 (50)		0	0	1 (100)	
Thoracic	22 (85)	4 (15)		1 (25)	3 (75)	0)	
Abdominal	20 (83)	4 (17)		0	4 (100)	0	
Pelvic	5 (100)	0		0	0	0	
**Bowel dysfunction**							
**Transverse degree of SCI**							
Tested patients (*n* = 54)	48 (88)	6 (12)	0.196*	1 (17)	5 (83)	0	1.000*
<33%	17 (100)	0		0	0	0	
33–66%	13 (81)	3 (19)		0	3 (100)	0	
>66%	18 (86)	3 (14)		1 (33)	2 (67)	0	
**Longitudinal intraspinal extension**							
Tested patients (*n* = 55)	51 (90)	6 (10)	0.066*	1 (17)	5 (83)	0	1.000*
1–3 vertebrae	24 (100)	0		0	0	0	
4–6 vertebrae	21 (81)	5 (19)		1 (20)	4 (80)	0	
>6 vertebrae	6 (86)	1 (14)		0	1 (100)	0	
**Intraspinal level**							
Tested patients (*n* = 55)	51 (89)	6 (11)	0.120*	1 (17)	5 (83)	0	1.000*
Cervical	1 (50)	1 (50)		0	1 (100)	0	
Thoracic	25 (96)	1(4)		0	1 (100)	0	
Abdominal	20 (83)	4 (17)		1 (25)	3 (25)	0	
Pelvic	5 (100)	0		0	0	0	
**Dyspnea**							
**Transverse degree of SCI**							
Tested patients (*n* = 187)	177 (94)	10 (6)	0.180*	2 (20)	2 (20)	6 (60)	0.119*
<33%	43 (100)	0		0	0	0	
33–66%	48 (92)	4 (8)		2 (50)	1 (25)	1 (25)	
>66%	86 (93)	6 (7)		0	1 (17)	5 (83)	
**Longitudinal intraspinal extension**							
Tested patients (*n* = 193)	183 (94)	10 (6)	0.833*	2 (20)	2 (20)	6 (60)	0.090*
1–3 vertebrae	56 (97)	2 (3)		2 (100)	0	0	
4–6 vertebrae	92 (94)	6 (6)		0	2 (33)	4 (67)	
>6 vertebrae	35 (95)	2 (5)		0	0	2 (100)	
**Intraspinal level**							
Tested patients (*n* = 193)	183 (95)	10 (5	<0.001*	2 (20)	2 (20)	6 (60)	0.714*
Cervical	7 (64)	4 (36)		0	1 (25)	3 (75)	
Thoracic	79 (93)	6 (7)		2 (33)	1 (17)	3 (50)	
Abdominal	80 (100)	0		0	0	0	
Pelvic	17 (100)	0		0	0	0	

* Fisher exact test.

Motor deficit was documented in 105 patients (80%) being the only SCI symptom in 35. It was more frequent in those younger than 36 months (85 vs. 54%; *P* = 0.001) and was rated severe in 46% of patients without age difference. The probability of motor deficit directly correlated with higher transverse degree (*P* < 0.001), greater longitudinal intraspinal extension (*P* = 0.001) and high intraspinal SCI level (*P* = 0.002). Its severity was directly correlated with degree (*P* = 0.019) and longitudinal extension (*P* = 0.037) of SCI (Table 6).

Pain was described in 69 patients (52%) and was the only SCI symptom in 11. It was more frequent (83 vs. 45%; *P* = 0.001) and more often severe (*P* = 0.003) in older patients and was less frequent in patients with <33% SCI degree (*P* = 0.031).

Spine deformities, mostly scoliotic changes, affected 30 patients (23%) without age difference. They were commonly associated with motor deficit and/or pain and more likely occurred in children with longitudinal SCI > 6 vertebrae (*P* 0.023).

Sensory deficit was reported in 21 patients (16%) and was always associated with other symptom(s) with no differences for age and imaging features.

Bladder and bowel functions were not considered evaluable in patients younger than 24 months although eight patients developed critical sphincter dysfunction(s) requiring urgent medical intervention. Among evaluable patients, bladder dysfunction was documented in 26% and bowel dysfunction in 18%, without difference by age. There was no correlation with neuroradiologic imaging features. Finally, dyspnoea was present in 10 patients (8%) and was equally distributed in the two age groups. It was the only SCI symptom in two patients and was only associated with cervical level (*P* < 0.001; Table 6).

## Discussion

In a Workshop on NB-SCI held in 2004, several experts reported contrasting results and agreed on the necessity to undertake cooperative studies (22). In 2014 the SIOPEN launched the first multinational prospective NB-SCI registry with the aim of collecting detailed information on a large number of SCI patients to be evaluated by standardized measures. With a data locked of July 2021, we have reported on the presenting characteristics of 196 NB-SCI patients and described the type and severity of symptoms in relation to imaging features.

Our prospective study is the largest cohort of children diagnosed with NB-SCI and confirms that one third of these patients are asymptomatic (7, 9, 12). We have demonstrated that asymptomatic patients have a less severe degree of SCI, fewer number of vertebrae involved and were less likely to undergo neurosurgical decompression (see Table 7 for comparison with previous reports).

**Table 7:** Presenting features of children enrolled in the NB-SCI study in comparison with previous studies.

**Feature**	**Punt** **et al. (4)**	**Plantaz et al. (8)**	**De Bernardi et al. (9)**	**Katzenstein et al. (10)**	**Simon et al. (11)**	**De Bernardi et al. (12)**	**Fawzy et al. (13)**	**This study 2022**
No. of NB pts	NR	315	1,462	NR	2,063	571 infants	576	NR
No. of NB pts with SCI (% of total NBs)	21	42 (13%)	76 (5.2%)	83	122 (4.7%)	43 infants (7.5%)	51 (9%)	196
No. of NB pts with symptomatic SCI (% of pts with SCI)	21 (100)	27 (64)	76 (100)	43 (52)	122 (100)	34 (79)	34 (67)	132 (67)
Male/female ratio	2.0	NR	1.0	NR	1.0	1.4	1.0	0.8
Median age at diagnosis, months	12	8	16	10	9	3	32	11
Median interval between symptoms and diagnosis, weeks	4	3	8	NR	NR	2	NR	2
Localized disease stage, %	100	100	71	75	92	85	49	66
Thoracic primary, %	62	40	37	66	39	38	31	49
Benign histopathology, %	22	38	11	NR	74*	NR	29*	11
Amplified *MYCN*, %	NR	3	NR	5	5	3	8	8
**Symptoms, %**								
Motor deficit	100	64	98	NR	95	85	59	80
Pain	30	NR	71	NR	56	38	12	52
Sensory	NR	NR	24	NR	58	NR	NR	16
Spine	NR	NR	NR	NR	NR	NR	NR	16
Both sphincter (bladder/bowel)	43	29	35	NR	(44/43)	21	(9/6)	(26/18)
Dyspnea	NR	NR	NR	NR	NR	3	NR	8
Unspecified	-	-	-	98	-	-	-	-

NR, not reported.

* Reported as favorable Shimada histology.

In agreement with previous NB-SCI studies (8, 10, 12), the presenting features of our patients differed from those expected in an overall NB population (11, 30, 31) for several characteristics: younger age (median, 11 vs. 16–19 months), greater frequency of thoracic primary tumors (49 vs. 20–30%) and localized disease (66 vs. 44–53%), and lower proportion of MYCN gene amplification (7 vs. 15–20%). Whereas, the high frequency of thoracic tumors in NB-SCI is explained by the fact that all thoracic NBs arise in the paravertebral ganglia whereas two thirds of the abdominal tumors arise in the adrenal gland, the reasons for the other discrepancies remain elusive.

T2 hyperintensity of spinal cord, consistent with oedema secondary to compression (29) occurred with greater frequency in symptomatic vs. asymptomatic patients, although the difference was not significant. The impact of T2 hyperintensity on functional outcome will be analyzed in more details in the ongoing follow-up study.

The median interval between the first symptoms and NB-SCI diagnosis was 14 days, comparable to some (10, 11), but shorter than others reports (8, 9, 14, 16, 17). An interval >60 days was observed in 12% of cases and occurred more frequently in patients with benign histology, likely reflecting the slow growth rate of these tumors (32). The interval between the first medical visit and diagnosis, not reported in previous studies, was remarkably short with a median value of 3 days. Overall, these figures suggest general awareness of SCI symptoms and easy access to MRI/CT imaging throughout the SIOPEN community.

Thanks to the standardized prospective data collection and the correlation with imaging features, our study provides detailed information on the characteristics of NB-SCI patients. We confirm that motor deficit affects the large majority of SCI symptomatic patients but also demonstrated its association with high intra-spinal level, larger transverse degree and, for the first time, greater longitudinal extension of SCI. As in other series (4, 8–11), almost half our SCI patients presented with the grade 3 motor deficit (paraplegia) despite the short intervals registered between initial SCI symptoms, first medical visit and diagnosis. Therefore, it appears unlikely that shortening the interval between onset of symptoms and diagnosis translate into fewer paraplegias.

Pain was described in 52% of patients, a figure comparable to 54 and 56% of the Italian (9) and German series (11). We were the first to quantify pain using standardized score for age, i.e., the FLACC score in preverbal children and CTCAE for older patients. In doing so, we observed a higher frequency among the older patients (83 vs. 45%). We believe that, in addition to age related differences in vocalizing and localizing pain, the small cross sectional area of the vertebral canal of younger children may account for motor deficit being the first SCI symptom, while the larger vertebral canal area of older children may allow pain to be the initial symptom before cord compression leads to motor deficit.

Spinal deformities, scarcely reported at onset in other NB-SCI series, involved 23% of our cases. Due to the short time interval between symptoms and diagnosis, this likely represents a secondary effect of motor deficit and/or pain rather than an autonomous symptom.

Sensory deficit was reported in 16% of patients with no correlation with age or imaging features. Its frequency is comparable with the Italian series (9), but differs from the 58% reported in the German study (11) and only one case found in the 52 patients of the North American series (10), suggesting that the reporting of sensory deficit should be further implemented and standardized.

Previous NB-SCI studies evaluated bladder and bowel dysfunctions as an aggregate symptom without an age cut-off and reported frequencies ranging from 14 to 52% (8, 9, 11, 14). In our series we deliberately excluded younger children because their sphincter function is poorly controlled and difficult to evaluate. Consequently, bladder/bowel function was assessed in <20% of all patients. Bladder and/or bowel dysfunction were found in 26 and 18%, respectively. In our series, bladder dysfunction (nine cases) was the third most frequent symptom (24%) among symptomatic patients older than 24 months. We suggest that age stratification should be regularly considered in future studies.

Dyspnoea, a symptom occasionally identified in previous series, was present in 8% of the cases and was significantly associated with the cervical SCI level. This finding was expected as the main respiratory muscles are innervated by phrenic neurons located in the cervical spinal cord.

## Conclusion

In conclusion, this SIOPEN study represents the first prospective multinational registry on NB-SCI. The precise data collection provided detailed information on SCI presenting features and differences with general NB populations, in part confirming previous retrospective studies. This study reports new information regarding intervals between SCI symptoms, medical referral and imaging, and provides correlations between clinical characteristics of NB-SCI and related imaging features.

## Data availability statement

The raw data supporting the conclusions of this article will be made available by the authors, without undue reservation.

## Ethics statement

The study was approved by the Ethics Committee of participating institutions and/or national NB groups. All participants or their parents/legal guardians gave consent to participate in this study.

## Author contributions

SS, SA, RH, DP, DK, BH, AW, KK, MB, BD, and TT: conception and design. IS, TSi, TSh, WB, CT, and GL: provision of data material or patients. FS and AL: collection and interpretation of patients' imaging. GE and MF: data analyses. SS, SA, RH, BD, and TT: manuscript writing. All authors: final approval of manuscript and accountability for all aspects of the work.

## Funding

This study was partially supported by the Italian Ministry of Health, Ricerca Corrente 2022, no grant applicable, Fondazione Italiana per la lotta al Neuroblastoma, and Children's Cancer Foundation: Project 305: SIOPEN Neuroblastoma Clinical Trials in Australia and New Zealand.

## Conflict of interest

The authors declare that the research was conducted in the absence of any commercial or financial relationships that could be construed as a potential conflict of interest.

## Publisher's note

All claims expressed in this article are solely those of the authors and do not necessarily represent those of their affiliated organizations, or those of the publisher, the editors and the reviewers. Any product that may be evaluated in this article, or claim that may be made by its manufacturer, is not guaranteed or endorsed by the publisher.

## Appendix

### Contributing institutions

Australia: Monash Children's Hospital, Melbourne, Perth Children's Hospital, Perth and Sydney Children's Hospital, Sydney. Austria: Hospital of Graz. Belgium: Hospitals of Bruxelles, Leuven and Antwerp. France: Hospitals of Amiens, Angers, Bordeaux, Brest, Grenoble, Lyon, Marseille, Nancy, Nantes, Paris, Poitiers, Reims, Rouen, St. Etienne, Strasbourg, Tours, Villejuif. Germany: Hospitals of Berlin, Bielefeld, Cottbus, Datteln, Dortmund, Duesseldorf, Erfurt, Erlangen, Frankfurt, Giessen, Heidelberg, Homburg/Saar, Kiel, Magdeburg, Munster, Nordhauser, Sankt Augustin, Stuttgart, Ulm. Ireland: Hospital of Dublin. Israel: Hospitals of Petach-Tiqva, Haifa, Tel Aviv. Italy: Hospitals of Bari, Genova, Bergamo Catania, Milano Modena, Bergamo, Napoli, Palermo, Parma, Roma, Torino and Trieste. Norway: Hospital of Trondheim. Poland: Hospitals of Chorzow, Rzeszow, Krakow and Wroclaw. Portugal: Hospital of Lisboa. Russia: Federal Scientific and Clinical Center of Moscow. Spain: Hospital of Valencia. Sweden: Hospitals of Linkoping, Lund and Stockholm. Switzerland: Hospital of Lausanne. The Netherlands: Prinses Maxima Center of Utrecht.
